# Tumor Microenvironment-Responsive Polymeric iRGD and Doxorubicin Conjugates Reduce Spontaneous Lung Metastasis in an Orthotopic Breast Cancer Model

**DOI:** 10.3390/pharmaceutics14081725

**Published:** 2022-08-18

**Authors:** Zheng-Hong Peng, Chinmay M. Jogdeo, Jing Li, Ying Xie, Yazhe Wang, Yuri M. Sheinin, Jindřich Kopeček, David Oupický

**Affiliations:** 1 Center for Drug Delivery and Nanomedicine, Department of Pharmaceutical Sciences, College of Pharmacy, University of Nebraska Medical Center, Omaha, NE 69198, USA; 2 Department of Pharmaceutics and Pharmaceutical Chemistry/CCCD, Department of Biomedical Engineering, University of Utah, Salt Lake City, UT 84112, USA; 3 Department of Pathology, Medical College of Wisconsin, 9200 W. Wisconsin Avenue, Milwaukee, WI 53226, USA

**Keywords:** breast cancer, cancer metastasis, iRGD, doxorubicin, splenomegaly, hematopoiesis

## Abstract

Tremendous progress has been made in the field of nanomedicine for cancer treatment. However, most of the research to date has been focused on inhibiting primary tumor growth with comparatively less efforts directed towards managing tumor metastasis. Here, we introduce a polymeric conjugate P-DOX-iRGD that not only significantly suppressed primary tumor growth but also substantially inhibited pulmonary metastasis in an orthotopic mouse model of breast cancer. In addition, treatment with P-DOX-iRGD markedly reduced breast cancer-induced splenomegaly and liver hematopoiesis. Interestingly, contrasting results were seen for the free form and polymeric form of DOX in vitro and in vivo, which may be attributed to the enhanced permeability and retention (EPR) effect.

## 1. Introduction

Breast cancer is the second most common cause of cancer-related death in American women, and over 90% of breast cancer-related death is due to metastatic breast cancer. Cancer metastasis is a complex, multistage process that includes the following steps: (i) escape of cancer cells from the primary tumor; (ii) circulation and survival of the cells in the blood stream and/or lymph nodes; (iii) extravasation; and (iv) proliferation and metastasis of the cancer cells to form secondary tumors at distant organs [1,2,3]. Surgical and radiation therapies are effective in resecting primary tumors, but are not effective in removing distant metastases. Similarly, chemotherapeutics including doxorubicin (DOX), which is one of the most common treatments for metastatic breast cancer [4], have little to no effect in inhibiting metastasis. This scarcity of drugs to combat metastasis prompted us to develop novel therapeutics against cancer metastasis.

Integrins, a family of heterodimeric cell surface receptors, play critical roles in the progression and metastasis of breast cancer [5,6]. Clinically, integrin α_ν_β_3_ is one of the most important indicators for breast cancer metastasis, since its expression in patients with metastasis is much higher than in those without metastasis [7]. The arginine-glycine-aspartic acid (RGD) sequence in the newly developed tumor-penetrating peptide iRGD (cyclic CRGDKGPDC) is an important binding motif for α_ν_ integrins. Proteolytic processing of iRGD exposes the neurophilin-1 binding RGDK sequence, which can activate an endocytic transport pathway through the tumor tissue. Once activated, this pathway can then take iRGD bound and unbound payloads deep in the tumor tissue, making iRGD both a tumor-homing and a tumor-penetrating peptide [8,9]. However, the short half-life of free iRGD due to proteolysis and rapid renal clearance limits it use in the clinic. iRGD conjugation to larger polymers helps overcome this limitation [10].

Nanotherapeutics including polymer conjugates take advantage of improved circulation times via the enhanced permeability and retention (EPR) effect to improve tumor penetration and accumulation. However, the efficiency of the EPR effect is often compromised due to heterogeneity of the tumor microenvironment, such as the abundant stroma, poor blood flow, and increased interstitial fluid pressure [11]. Using targeting moieties such as iRGD is one of the strategies to enhance the tumor targeting and penetration ability of nanomedicines, consequently improving the treatment efficacy [12]. Combination therapies, which involve the co-delivery of drugs with different mechanisms, are emerging as an attractive strategy for cancer treatment. Due to ease of functionalization, nanomaterials provide a perfect carrier for combinational drug delivery. Here, we report on the development of a polymer conjugate, combining two drugs for inhibiting primary tumor growth and metastasis in a 4T1 orthotopic breast cancer model.

Recently, we reported the development of a novel polymer P-DOX-iRGD, in which iRGD and DOX were conjugated to the hydrophilic, non-immunogenic N-(2-hydroxypropyl) methacrylamide (HPMA) copolymer via two different cleavable spacers [13]. Doxorubicin (DOX) was conjugated to the HPMA copolymer by a lysosomally cleavable tetrapeptide (GFLG), and an MMP-2-degradable linker (PLGLAG) connected the iRGD peptide to HPMA. Results from the previous study indicated that the covalent conjugation of iRGD via MMP2-sensitive bonds enhanced the accumulation and penetration of the conjugate into tumor cell monolayers and spheroids. Prolonged treatment with the iRGD peptide initiated before metastasis development has recently been shown to inhibit spontaneous breast cancer metastasis with little to no effect on the growth of the primary tumor [9,14]. Similarly, the considerable potential of iRGD in metastasis prevention has been demonstrated in several other cancer types [15]. In this study, the efficacy of this polymer conjugate was examined in an orthotopic 4T1 metastatic breast cancer model. We hypothesized that the cleavable conjugation of iRGD and DOX to the polymer will lead to a combination therapeutic effect attributable to DOX and iRGD, resulting in the inhibition of breast cancer growth as well as metastasis.

## 2. Materials and Methods

### 2.1. Materials

Organic solvents were purchased from Fisher Scientific (Hampton, NH, USA). Dulbecco’s Modified Eagle Medium (DMEM), L-glutamine, fetal bovine serum (FBS), sodium pyruvate, essential amino acids, and non-essential amino acids were obtained from Hyclone (Logan, UT, USA). Bouin’s solution was purchased from Sigma-Aldrich (St. Louis, MO, USA). The 4T1 mouse mammary carcinoma cell line was obtained from the ATCC and grown in DMEM medium supplemented with 10% FBS, 2 mM L-glutamine, 1 mM sodium pyruvate, 2% essential amino acids, 1% non-essential amino acids, and 0.4% gentamycin at 37 °C in a humidified atmosphere of 5% CO_2_. All other reagents were purchased from Fisher if not mentioned otherwise.

### 2.2. Preparation of Polymer Conjugate P-DOX-iRGD

**Figure 1:**
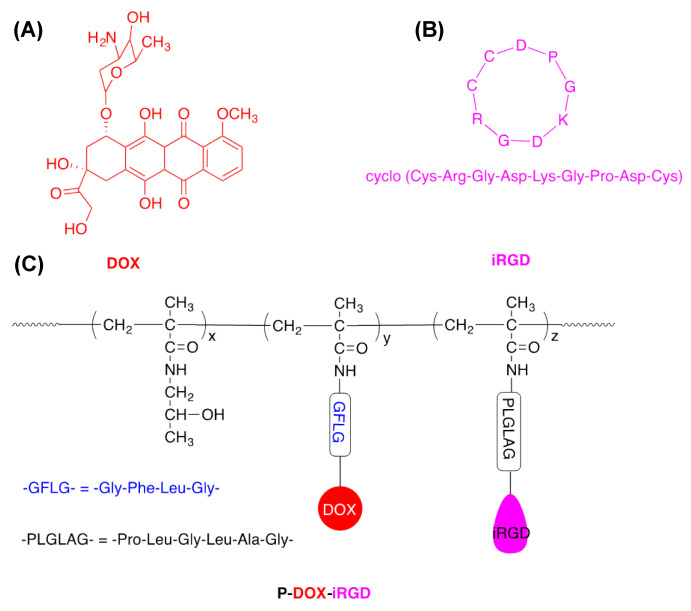
Chemical structures of (**A**) DOX, (**B**) RGD, and (**C**) P-DOX-iRGD.

The structures of iRGD, DOX, and P-DOX-iRGD are shown in Figure 1. The polymer P-DOX-iRGD was synthesized as previously reported [13]. Briefly, P-DOX-iRGD was synthesized by copolymerization of monomers HPMA, MA-GFLG-DOX, and MA-GG-PLGLAG-iRGD. The molecular weight and molecular weight distribution of the polymer were measured on AKTA FPLC. The molecular weight and molecular weight distribution of P-DOX-iRGD was measured on a Superose 6 HR10/30 analytical column. Sodium acetate (0.1 M) in a mixture of 30% acetonitrile/70% DI water (*v*/*v*) (pH = 6.5) was used as the elute, and the flow rate was 1 mL/min. The iRGD content in the polymer was measured by Amino Acid Analysis. The iRGD content in conjugate P-DOX-iRGD was determined by amino acid analysis. The first step was to hydrolyze the peptide by dissolving 2.5 mg of conjugate P-DOX-iRGD in 0.5 mL of 6 M HCl and heating at 110 °C for 24 h. The solvent was removed under reduced pressure and the residue was redissolved in 100 μL of DI water. Then, the hydrolyzed amino acid was derivatized by sequential addition: 20 μL of potassium tetraborate in distilled water (150 μg/μL), 20 μL of o-phthaldialdehyde in methanol (50 μg/μL), and 20 μL of mercaptopropionic acid in distilled water (0.05 mL/1 mL). The fluorescence (Ex = 229 nm, Em = 450 nm) of the hydrolyzed amino acid derivatives was measured by analytical HPLC (Agilent Technologies 1100 series, XDB-C8, 5 μm, column 4.6 × 150 mm) using gradient elution with buffers A and B (Buffer A: 0.05 M sodium acetate in 25 mL of acetonitrile and 975 mL of DI water, pH 6; Buffer B: 0.05 M sodium acetate in 300 mL of DI water and 700 mL of methanol, pH 6), and the flow rate was 1.0 mL/min. Aspartic acid (1 mM, 2.5 mM, 5 mM) and alanine (0.3 mM, 1 mM, 3 mM) were used for calibration. The content of DOX in the polymer was measured by UV spectrometry.

### 2.3. Cellular Uptake

4T1 cells were grown on 6-well plates (1 × 10^5^ cells/well) for 24 h and then incubated with 1 mL fresh media containing free iRGD (0.9 μM), free DOX (1 μM), or free iRGD (0.9 μM) plus free DOX (1 μM) or the polymeric conjugate P-DOX-iRGD [(0.9 μM iRGD) and (1 μM DOX) equivalents] for 4 h. The cells were then washed with phosphate buffered saline (PBS), detached from the plates using TrypLE^TM^, resuspended in PBS, and analyzed using BD FACSCalibur™ flow cytometer. The data were analyzed using FlowJo software. For cell uptake by confocal microscopy, 4T1 cells were grown on four-chamber coverslips (Thermo Fisher Scientific, Waltham, MA, USA) (5 × 10^3^ cells in 500 μL media per chamber) for 24 h. The old media was replaced with 100 µL media containing free iRGD (0.9 μM) or free DOX (1 μM), free iRGD (0.9 μM) plus free DOX (1 μM), or polymeric conjugate P-DOX-iRGD [(0.9 μM iRGD) and (1 μM DOX) equivalent] and incubated for 4 h. The drug solutions were removed, and cells were washed with PBS. The images were acquired with Olympus FV1000-IX81 confocal microscope (excitation at 488 nm, emission collected with a 515 nm barrier filter). The data were processed with ZEN lite software.

### 2.4. In Vitro Cytotoxicity

The cytotoxicity of DOX conjugates and controls in 4T1 cells was measured by CellTiter-Blue^®^ Cell Viability Assay (Promega, Madison, WI, USA). 4T1 cells were seeded in 96-well plates at a density of 10,000 cells/well in 100 µL enriched DMEM media containing 10% FBS. After 6 h, the culture media were replaced with 100 µL media or different concentrations of DOX, DOX plus iRGD, and polymeric conjugate ([DOX] equivalent). The mol ratio of [iRGD]/[DOX] was 9/10. After 72 h, the cell culture media were replaced with 120 µL of 1:5 diluted CellTiter-Blue solution and incubated for 1–2 h. The fluorescence of the reduced product resorufin was measured with a SpectraMax M Series Multi-Mode Microplate reader (Molecular Devices, Sunnyvale, CA, USA). The data were analyzed using GraphPad Prism v. 5.03 (GraphPad Software Inc., La Jolla, CA, USA).

### 2.5. In Vivo Anti-Tumor Efficacy

All animal studies were conducted in compliance with the Institutional Animal Care and Use Committee (IACUC) guidelines in the University of Nebraska Medical Center. Female BALB/c mice bearing 4T1 tumors (8 mice per group) were chosen to investigate the therapeutic effect of free iRGD, DOX, free iRGD plus DOX, and the polymer conjugate P-DOX-iRGD for inhibiting breast cancer growth and metastasis. The orthotopic 4T1 breast cancer mouse model was established by injecting 1 × 10^4^ 4T1 cells in the right flank of female BALB/c mice (6–8 weeks old, 15–25 g), purchased from the Charles River Laboratories Inc. (Wilmington, MA, USA). When the tumor volume reached approximately 50 mm^3^, the mice were randomly divided into five groups and received four injections (days 8, 10, 12, and 14) as follows: (i) untreated, (ii) free iRGD (7.8 mg/kg), (iii) DOX (5 mg/kg), (iv) a combination of iRGD (7.8 mg/kg) and DOX (5 mg/kg), and (v) polymeric conjugate (P-DOX-iRGD) (7.8 mg/kg iRGD equivalent and 5 mg/kg DOX equivalent). Body weights and tumor volumes were monitored every other day. The timepoint when the tumor weight reached 10% of the pre-dosing weight in the untreated mice was considered as the study end point and the mice were euthanized. Tumors and major organs (spleen, liver, kidney, and heart) were excised and stored in 4% formalin. The lungs were kept in Bouin’s solution (Sigma-Aldrich) for 24 h and the metastases on the lung surface were visualized under a microscope. After counting the surface metastases, the lungs were stored in 4% formalin. The organs were embedded in paraffin, sectioned, and stained with hematoxylin and eosin (H&E) for histological analysis.

### 2.6. Statistical Analysis

Data are presented as mean ± standard deviation. The differences among multiple groups were analyzed by one-way ANOVA. The statistical significance between the two groups were analyzed by using a Student’s *t*-test, where *p <* 0.05 was considered as the minimal level of significance. All statistical analyses were performed with GraphPad Prism.

## 3. Results

### 3.1. Accumulation of DOX in the 4T1 Tumor Cells

**Figure 2:**
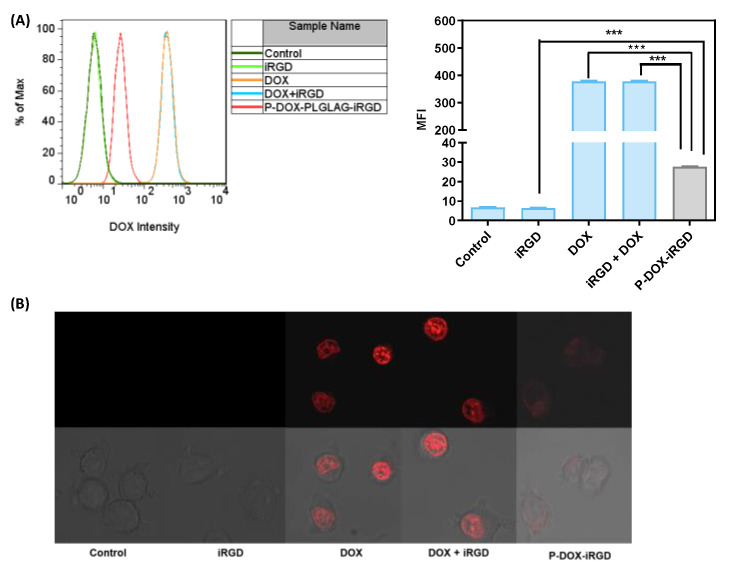
Accumulation of DOX conjugates and controls in 4T1 cells as determined by DOX fluorescence: (**A**) Flow Cytometry, (**B**) Confocal Microscopy. Data are expressed as mean ± SD, *** *p* < 0.001.

4T1 mouse mammary carcinoma cells were used for in vitro studies. 4T1 cells are very sensitive to DOX; hence, lower concentrations of DOX and iRGD equivalents compared to the previously published data were used in this study. First, the accumulation of polymer P-DOX-iRGD and related controls (iRGD, DOX, iRGD plus DOX) in 4T1 mammary carcinoma cells was investigated by measuring the intrinsic fluorescence of DOX with flow cytometry and confocal microscopy (Figure 2A,B). The cells treated with free DOX or a combination of DOX and iRGD showed about 12-fold higher uptake than the polymer P-DOX-iRGD. These results indicate that in vitro, 4T1 cells show a higher uptake of the free form of DOX than the polymeric form. The addition of free iRGD did not enhance the uptake of free DOX. The uptake data were validated using confocal microscopy. The images show that the red fluorescence from DOX in the free DOX and iRGD plus DOX-treated cells was much more intense than that of P-DOX-iRGD, indicating a higher uptake of free DOX in the cells.

### 3.2. In Vitro Cytotoxicity

**Figure 3:**
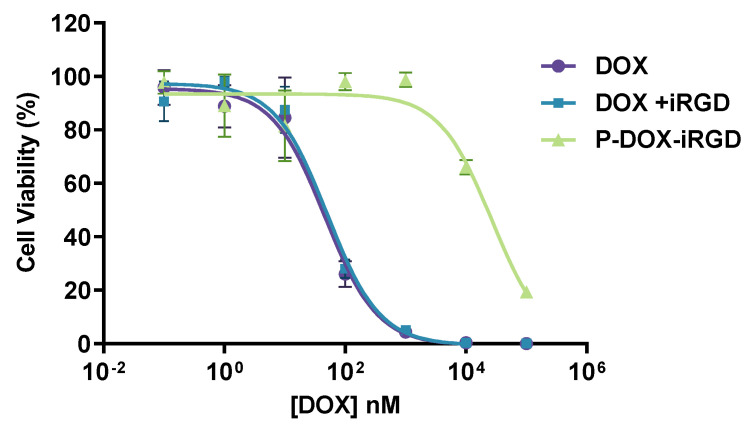
Cytotoxicity of the formulations in 4T1 cells.

In vitro cytotoxicity of polymeric conjugate (from 0.1 nM to 100 µM of DOX equivalent) and related controls was determined using the CTB assay. The mol ratio of [iRGD]/[DOX] was 9/10, based on the mol ratio of iRGD/DOX in the polymer conjugate. The cell viability percent versus DOX concentration are summarized in Figure 3. The IC_50_ values of DOX, DOX plus iRGD, and P-DOX-iRGD were 46.4 nM, 50.7 nM, and 26.6 µM. These results indicate that in agreement with the cellular uptake data, the polymeric conjugate P-DOX-iRGD was significantly less toxic than free DOX. The combination of DOX and iRGD had similar cytotoxicity as free DOX indicating that free iRGD had a negligible effect on enhancing the cytotoxicity of DOX toward 4T1 cells.

### 3.3. Inhibition of 4T1 Primary Tumor Growth

**Figure 4:**
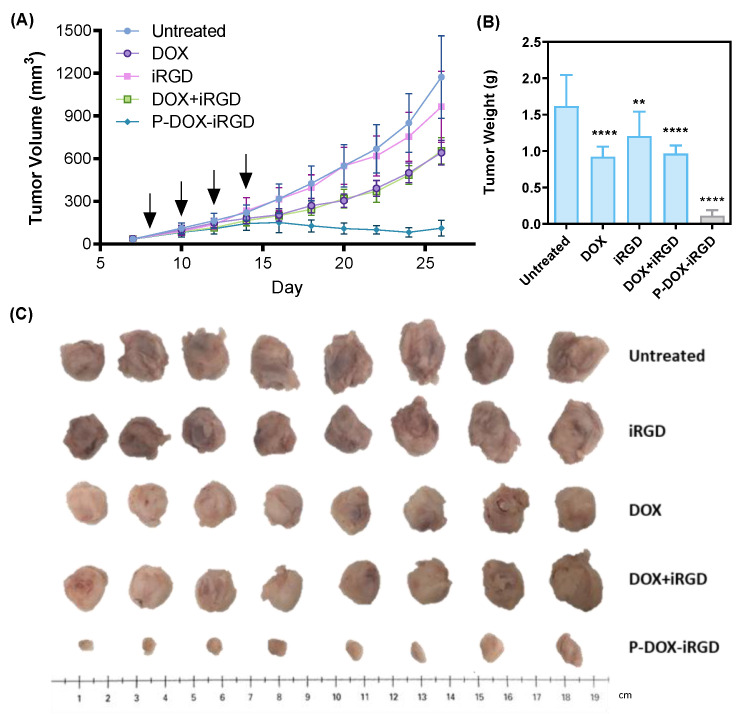
Anticancer activity of the polymer conjugate in an orthotopic 4T1 breast cancer model in BALB/c mice. (**A**) Tumor volume during treatment. (**B**) Primary tumor weight on day 26. (**C**) Primary tumor images on day 26. Data are shown as mean ± SD (*n* = 8) ** *p* < 0.01, **** *p* < 0.0001. Arrows indicate the days of treatment injections (days 8, 10, 12, and 14).

The antitumor efficacy of P-DOX-iRGD was tested in a murine orthotopic 4T1 breast cancer model. This is a clinically relevant model for studying breast cancer metastasis resembling advanced breast cancer in women because the 4T1 cells can spontaneously metastasize from the primary tumor in the mammary gland to distant sites [16]. First, we assessed the capacity of DOX, iRGD, DOX plus iRGD, and P-DOX-iRGD to inhibit the growth of primary tumor by measuring the change in tumor volume, weight, and size (Figure 4A–C) after treatment. At the end study date (day 26), in comparison to the untreated group, treatment with iRGD showed negligible effect on primary tumor growth, confirming that free iRGD has no significant effect on tumor cell proliferation. Both DOX and iRGD plus DOX inhibited primary tumor grown by ~50%. Treatment with the polymeric conjugate P-DOX-iRGD showed significant anticancer activity, with about 90% inhibition of primary tumor growth. 

### 3.4. Reduction of Breast Cancer Pulmonary Metastasis

**Figure 5:**
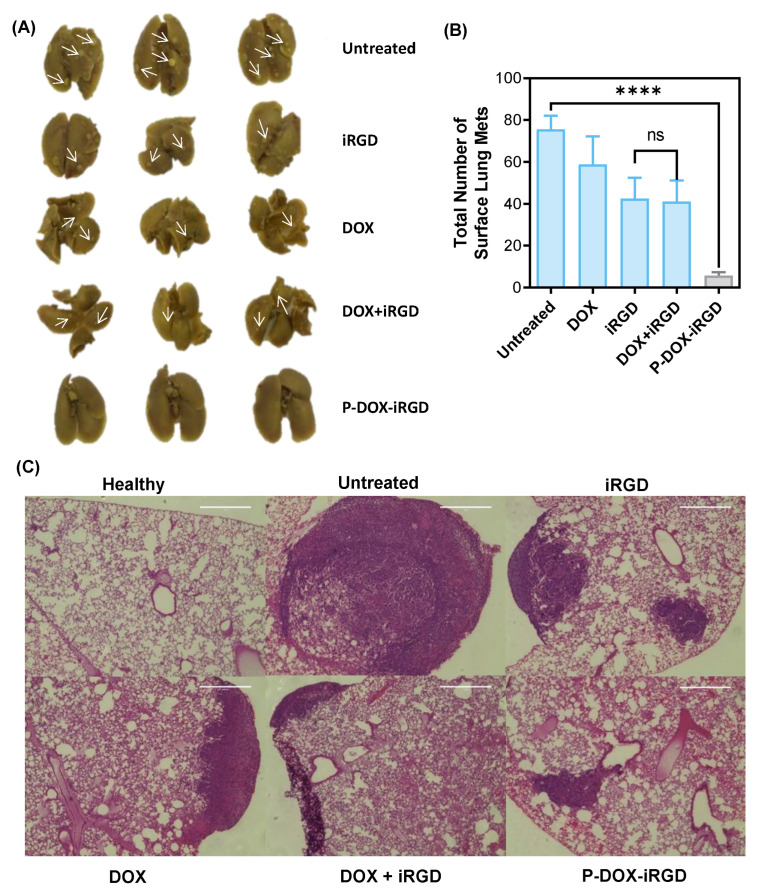
Reduction of breast cancer pulmonary metastasis. (**A**) Representative images of lungs. (**B**) Number of pulmonary metastatic foci. (**C**) H&E-stained lungs metastases from 4T1 tumor-bearing mice. Data are expressed as mean ± SD. **** *p* < 0.0001. Scale bar = 100 μm.

Breast cancer can metastasize from the primary tumor site to distant organs, especially lung, liver, bone, and brain, through blood vessels and lymphatic channels [17,18]. The overall survival of patients with pulmonary metastasis (18.0 months) is the poorest when compared to metastasis at other sites (brain, 26.9 months; liver, 38.2 months; bone, 50.8 months) [19]. Images of lungs from mice with breast cancer are shown in Figure 5A, the white spots indicated by arrows are the metastases. The average number of lung metastases (Figure 5B) in the P-DOX-iRGD group was significantly lower compared to other groups indicating that treatment with the polymer substantially inhibited lung metastasis. The difference between iRGD- and DOX plus iRGD-treated groups was not significant. However, both groups had lower instances of lung metastases as compared to free DOX. This indicated iRGD plays a critical role in inhibiting the breast cancer metastasis. The polymeric conjugate P-DOX-iRGD markedly inhibited metastasis compared to the other groups. The histochemical analysis of the H&E-stained lung (Figure 5C) further confirmed the significance of P-DOX-iRGD in inhibiting the breast cancer pulmonary metastasis. Overall, treatment with P-DOX-iRGD significantly reduced the number and size of detectable breast cancer lung metastatic foci.

### 3.5. Inhibition of Tumor-Induced Splenomegaly and Liver Hematopoiesis

**Figure 6:**
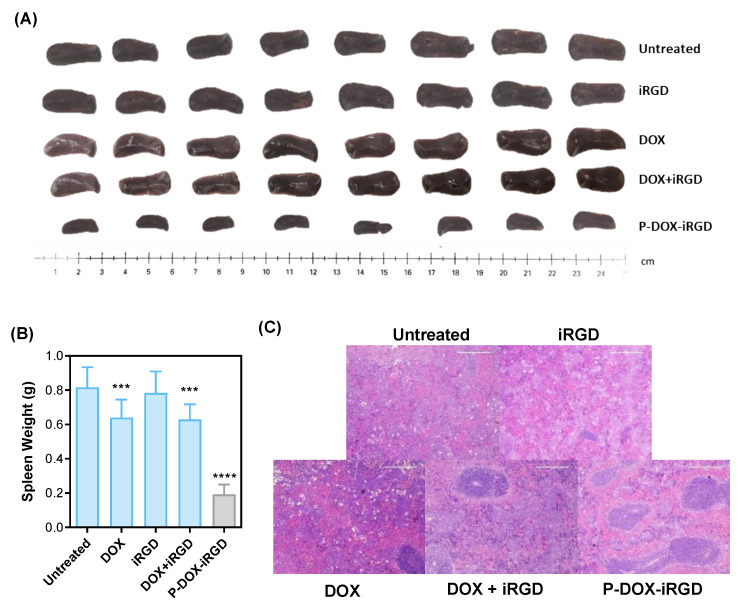
Reduction of tumor-induced splenomegaly. (**A**) Representative images of spleens on day 26. (**B**) Weight of spleens on day 26. (**C**) Hematoxylin and eosin stains (H&E) of spleens. Data are expressed as mean ± SD *** *p* < 0.001, and **** *p* < 0.0001 Scale bar = 200 μm.

Splenomegaly (enlarged spleen), which results from the expansion of splenic granulocytes including the myeloid derived suppressor cells (MDSCs), is observed in 4T1 tumor-bearing mice [20,21]. The images of spleens from mice at the study end-point are shown in Figure 6A. On day 26 after inoculation of 4T1 tumor cells, the spleen size in the untreated group was increased 8.5-fold compared to healthy mice. Treatment with free DOX, free iRGD, or a combination of free DOX and iRGD reduced the tumor-induced spleen enlargement by 24.6%, 4.56%, and 26.0%, respectively. In comparison, P-DOX-iRG—treated mice showed a significant 86.54% reduction in tumor-induced splenomegaly. These results indicate that the conjugation of DOX and iRGD to polymer significantly inhibited the 4T1 tumor-induced splenomegaly. This may partly be due to the suppression of MDSC accumulation in the spleen. It is well known that MDSCs are potent inhibitors of immune cells, and the decreased numbers of MDSCs in spleen is associated with the increased numbers of both T lymphocytes and B lymphocytes, thereby inhibiting tumor growth and development [20,22]. These results indicate that treatment with the polymeric conjugate P-DOX-iRGD could potentially enhance the anti-tumor immunological response. Histological analysis of the spleens (Figure 6C) revealed a significant reduction in white pulp and an increase in the red pulp of untreated mice bearing 4T1 tumors potentially caused by extramedullary hematopoiesis [20]. Treatment with iRGD had a negligible effect on inhibiting tumor-induced spleen hematopoiesis. On the other hand, treatment with P-DOX-iRGD significantly reduced the 4T1 breast tumor-induced spleen hematopoiesis. Similarly, extramedullary hematopoiesis and increased proportion of nucleated cells were also found in liver of untreated mice bearing 4T1 tumors in comparison to the liver from healthy mice (Figure S1) [23]. The iRGD, DOX, or iRGD + DOX-treated mice had reduced tumor-induced liver hematopoiesis. Treatment with P-DOX-iRGD almost completely inhibited the 4T1 tumor-induced liver hematopoiesis.

### 3.6. Safety Evaluation

It is essential to evaluate the potential in vivo toxicity of the polymeric conjugate. Body weight as a sign for acute toxicity was measured every other day. Body weight analysis of mice (Figure S2A) showed no significant weight loss or gain from any treatment. From day 16 to day 26, the weight of DOX-, DOX plus iRGD-, or P-DOX-iRGD-treated mice was slightly lower than that of other mice, which may be due to the shrinkage of tumors. Histological analysis of organs showed no significant morphological difference in kidneys (Figure S2B) and hearts (Figure S2C) after treatment, except for one tumor that was found in the kidney of the untreated mice bearing 4T1 tumor cells.

## 4. Discussion

Previously, we have reported the development of a novel polymer P-DOX-iRGD and demonstrated that the covalent conjugation of the iRGD peptide via MMP2-sensitive bonds enhanced the accumulation and penetration of the polymeric conjugate into tumor cell monolayers and spheroids. Since the anti-metastatic efficacy of the iRGD peptide has been demonstrated in several studies, we wanted to test the efficacy of this polymer in reducing spontaneous lung metastases in addition to inhibiting primary tumor growth in an orthotopic 4T1 breast cancer model. Hence, in this study, the polymeric conjugate was employed as a combination therapy which utilizes the efficacy of DOX against the primary tumors and tests the anti-metastatic efficacy of iRGD. We first assessed uptake of the polymer in 4T1 cells and found that the polymer had a lower uptake compared to free DOX. This low uptake then translated to a much lower in vitro cytotoxicity of the polymer in 4T1 cells in comparison to free DOX. In contrast to these in vitro results, P-DOX-iRGD significantly inhibited primary tumor growth in an orthotopic 4T1 breast cancer model when compared to free DOX. This discrepancy could possibly be attributed to enhanced tumor penetration and retention of the macromolecular polymeric conjugate due to the well documented EPR effect. In addition, the tumor-homing and -penetrating properties of the polymer due to iRGD could have played a role in the selective accumulation of the polymer at the primary tumor, leading to an enhanced anticancer effect. The iRGD peptide has anti-metastatic efficacy mediated through the binding of the CendR peptide motif to NRP-1 [9]. Here, we showed that the peptide demonstrated improved antimetastatic efficacy when conjugated to a polymer as demonstrated by the significantly reduced lung metastatic foci seen in the P-DOX-iRGD group in comparison to free iRGD when administered in equivalent doses. In addition, the 4T1 model is described to induce a leukemoid reaction leading to splenomegaly, which is associated with the systemic release of tumor-derived growth factors and metastasis [24]. The spleens of animals treated with the polymeric conjugate had significantly less splenomegaly with a near normal architecture in comparison to the other groups, which could further be a sign of decreased metastatic burden. This could possibly be attributed to the suppression of MDSCs accumulation in the spleen. However, a detailed study is needed to further elucidate the effect of P-DOX-iRGD on 4T1 tumor-induced splenomegaly. Thus, our results suggest that the iRGD peptide could be used as an adjuvant therapeutic to inhibit metastasis when used as a tumor targeting moiety, leading to an enhanced anticancer effect. In addition, the differences seen in our in vitro and in vivo studies provide further proof for the tremendous potential of the EPR effect in the targeted delivery of therapeutics to solid tumors.

## 5. Conclusions

In conclusion, the results presented here open new avenues for utilizing iRGD conjugated to polymeric systems as a therapeutic to treat metastasis in addition to its well documented tumor-homing and tumor-penetrating properties.

## Data Availability

Data is contained within the article or Supplementary Materials.

## Supplementary Materials

The following supporting information can be downloaded at: https://www.mdpi.com/article/10.3390/pharmaceutics14081725/s1, Figure S1: Hematoxylin and eosin stains (H&E) of Liver. Figure S2: (A) Average body weight change of tumor bearing mice. (B) Hematoxylin and eosin stains (H&E) of Kidneys. (C) Hematoxylin and eosin stains (H&E) of Heart.

## References

[1] Chaffer C.L., Weinberg R.A. (2011). A perspective on cancer cell metastasis. Science.

[2] Minn A.J., Gupta G.P., Siegel P.M., Bos P.D., Shu W., Giri D.D., Viale A., Olshen A.B., Gerald W.L., Massagué J. (2005). Genes that mediate breast cancer metastasis to lung. Nature.

[3] Wan L., Pantel K., Kang Y. (2013). Tumor metastasis: Moving new biological insights into the clinic. Nat. Med..

[4] Hassan M., Ansari J., Spooner D., Hussain S. (2010). Chemotherapy for breast cancer. Oncol. Rep..

[5] Desgrosellier J.S., Cheresh D.A. (2010). Integrins in cancer: Biological implications and therapeutic opportunities. Nat. Rev. Cancer.

[6] Cruet-Hennequart S., Maubant S., Luis J., Gauduchon P., Staedel C., Dedhar S. (2003). αv integrins regulate cell proliferation through integrin-linked kinase (ILK) in ovarian cancer cells. Oncogene.

[7] Gasparini G., Brooks P.C., Biganzoli E., Vermeulen P.B., Bonoldi E., Dirix L.Y., Ranieri G., Miceli R., Cheresh D.A. (1998). Vascular integrin alpha (v) beta3: A new prognostic indicator in breast cancer. Clin. Cancer Res..

[8] Gagnon M.L., Bielenberg D.R., Gechtman Z.e., Miao H.-Q., Takashima S., Soker S., Klagsbrun M. (2000). Identification of a natural soluble neuropilin-1 that binds vascular endothelial growth factor: In vivo expression and antitumor activity. Proc. Natl. Acad. Sci. USA.

[9] Sugahara K.N., Braun G.B., de Mendoza T.H., Kotamraju V.R., French R.P., Lowy A.M., Teesalu T., Ruoslahti E. (2015). Tumor-penetrating iRGD peptide inhibits metastasis. Mol. Cancer Ther..

[10] Pang H.-B., Braun G.B., She Z.-G., Kotamraju V.R., Sugahara K.N., Teesalu T., Ruoslahti E. (2014). A free cysteine prolongs the half-life of a homing peptide and improves its tumor-penetrating activity. J. Control. Release.

[11] Nichols J.W., Bae Y.H. (2014). EPR: Evidence and fallacy. J. Control. Release.

[12] Zhang B., Hu Y., Pang Z. (2017). Modulating the tumor microenvironment to enhance tumor nanomedicine delivery. Front. Pharm..

[13] Peng Z.-H., Kopeček J. (2015). Enhancing Accumulation and Penetration of HPMA Copolymer-Doxorubicin Conjugates in 2D and 3D Prostate Cancer Cells via iRGD Conjugation with an MMP-2 Cleavable Spacer. J. Am. Chem. Soc..

[14] Hamilton A.M., Aidoudi-Ahmed S., Sharma S., Kotamraju V.R., Foster P.J., Sugahara K.N., Ruoslahti E., Rutt B.K. (2015). Nanoparticles coated with the tumor-penetrating peptide iRGD reduce experimental breast cancer metastasis in the brain. J. Mol. Med..

[15] Zuo H. (2019). iRGD: A promising peptide for cancer imaging and a potential therapeutic agent for various cancers. J. Oncol..

[16] Pulaski B.A., Ostrand-Rosenberg S. (2001). Mouse 4T1 Breast Tumor Model. Current Protocols in Immunology.

[17] Weigelt B., Peterse J.L., van’t Veer L.J. (2005). Breast cancer metastasis: Markers and models. Nat. Rev. Cancer.

[18] Lee Y.T. (1983). Breast carcinoma: Pattern of metastasis at autopsy. J. Surg. Oncol..

[19] Gadiyaram V.K., Kurian S., Abraham J., Ducatman B., Hazard H., Hobbs G., Vona-Davis L. (2010). Recurrence and survival after pulmonary metastasis in triple-negative breast cancer. J. Clin. Oncol..

[20] DuPre S.A., Hunter K.W. (2007). Murine mammary carcinoma 4T1 induces a leukemoid reaction with splenomegaly: Association with tumor-derived growth factors. Exp. Mol. Pathol..

[21] Le H.K., Graham L., Cha E., Morales J.K., Manjili M.H., Bear H.D. (2009). Gemcitabine directly inhibits myeloid derived suppressor cells in BALB/c mice bearing 4T1 mammary carcinoma and augments expansion of T cells from tumor-bearing mice. Int. Immunopharmacol..

[22] Gabrilovich D.I., Nagaraj S. (2009). Myeloid-derived suppressor cells as regulators of the immune system. Nat. Rev. Immunol..

[23] Tao K., Fang M., Alroy J., Sahagian G.G. (2008). Imagable 4T1 model for the study of late stage breast cancer. BMC Cancer.

[24] Castro F., Pinto M.L., Pereira C.L., Serre K., Barbosa M.A., Vermaelen K., Gärtner F., Gonçalves R.M., De Wever O., Oliveira M.J. (2020). Chitosan/γ-PGA nanoparticles-based immunotherapy as adjuvant to radiotherapy in breast cancer. Biomaterials.

