# Supplementary material for: Tumor Microenvironment-Responsive Polymeric iRGD and Doxorubicin Conjugates Reduce Spontaneous Lung Metastasis in an Orthotopic Breast Cancer Model
Source: Pharmaceutics. 2022 Aug 18;14(8):1725. doi: 10.3390/pharmaceutics14081725 (PMC9416753; doi:10.3390/pharmaceutics14081725)
Supplement: Supplementary file 1 [file pharmaceutics-14-01725-s001.zip › pharmaceutics-1845416-supplementary.pdf]

Supporting Information

Tumor Microenvironment-Responsive Polymeric iRGD and Doxorubicin Conjugates Reduce Spontaneous Lung Metastasis in an Orthotopic Breast Cancer Model

Zheng-Hong Peng [1,2], Chinmay M. Jogdeo [1], Jing Li [1], Ying Xie [1], Yazhe Wang [1], Yuri M. Sheinin [1,3], Jindřich Kopeček [2,*] and David Oupický [1,*]

[1] Center for Drug Delivery and Nanomedicine, Department of Pharmaceutical Sciences, College of Pharmacy, University of Nebraska Medical Center, Omaha, NE 69198, USA;
[2] Department of Pharmaceutics and Pharmaceutical Chemistry/CCCD, Department of Biomedical
Engineering, University of Utah, Salt Lake City, UT 84112, USA
[3] Department of Pathology, Medical College of Wisconsin, 9200 W. Wisconsin Avenue, Milwaukee, WI 53226, USA
* Correspondence: jindrich.kopecek@utah.edu (J.K.); david.oupicky@unmc.edu (D.O.)

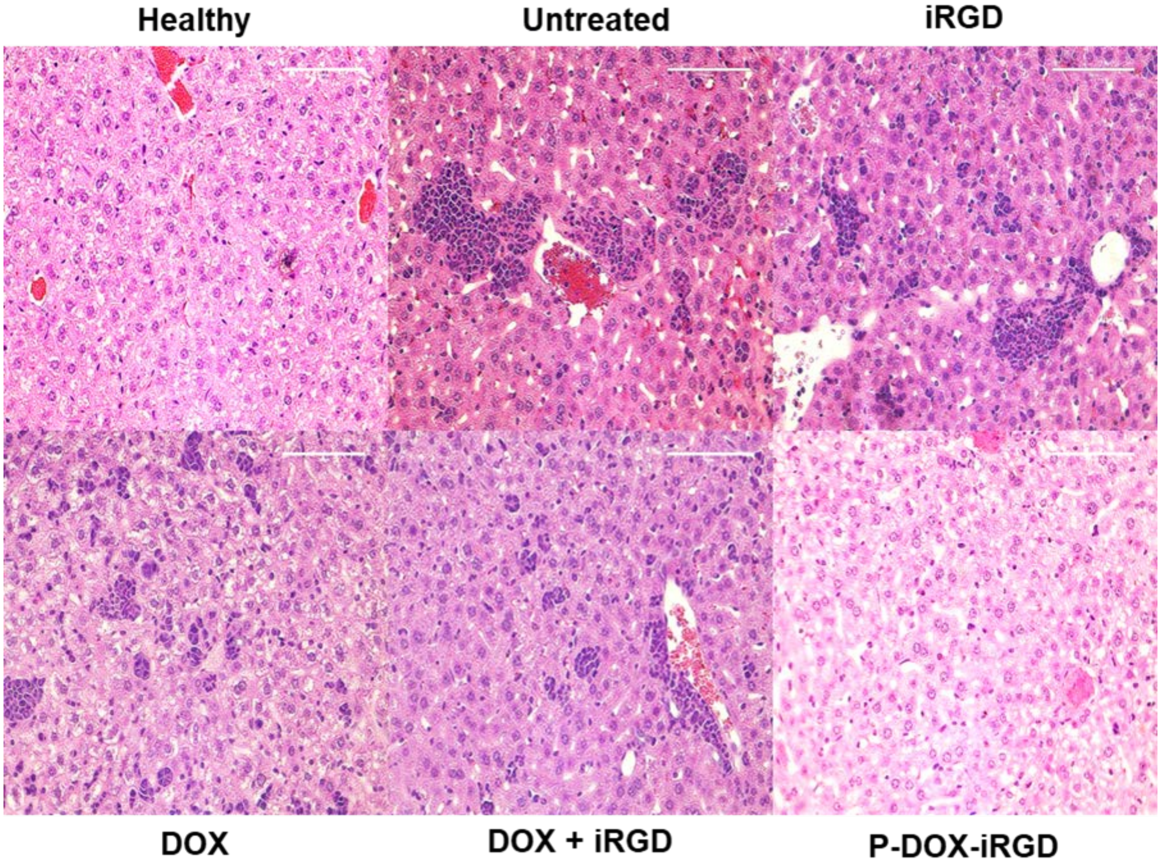

Figure S1. Hematoxylin and eosin stains (H&E) of liver. Scale bar = 100 μm

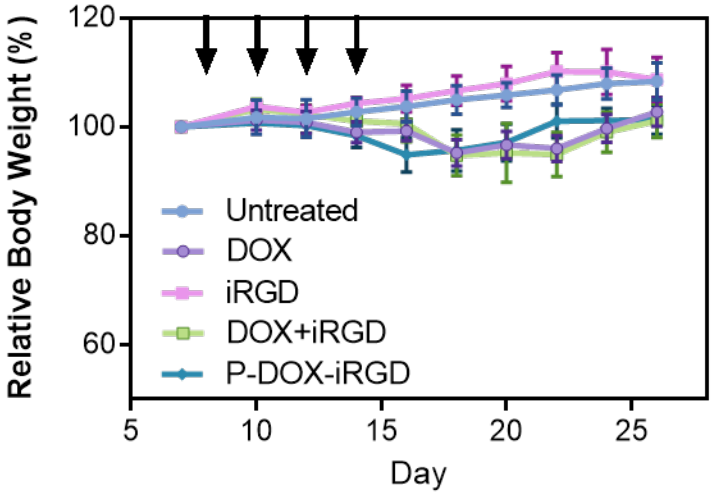

120
100
80
60
Relative Body Weight (%)
5
10
15
20
25
Day
Untreated
DOX
iRGD
DOX+iRGD
P-DOX-iRGD

(**A**)

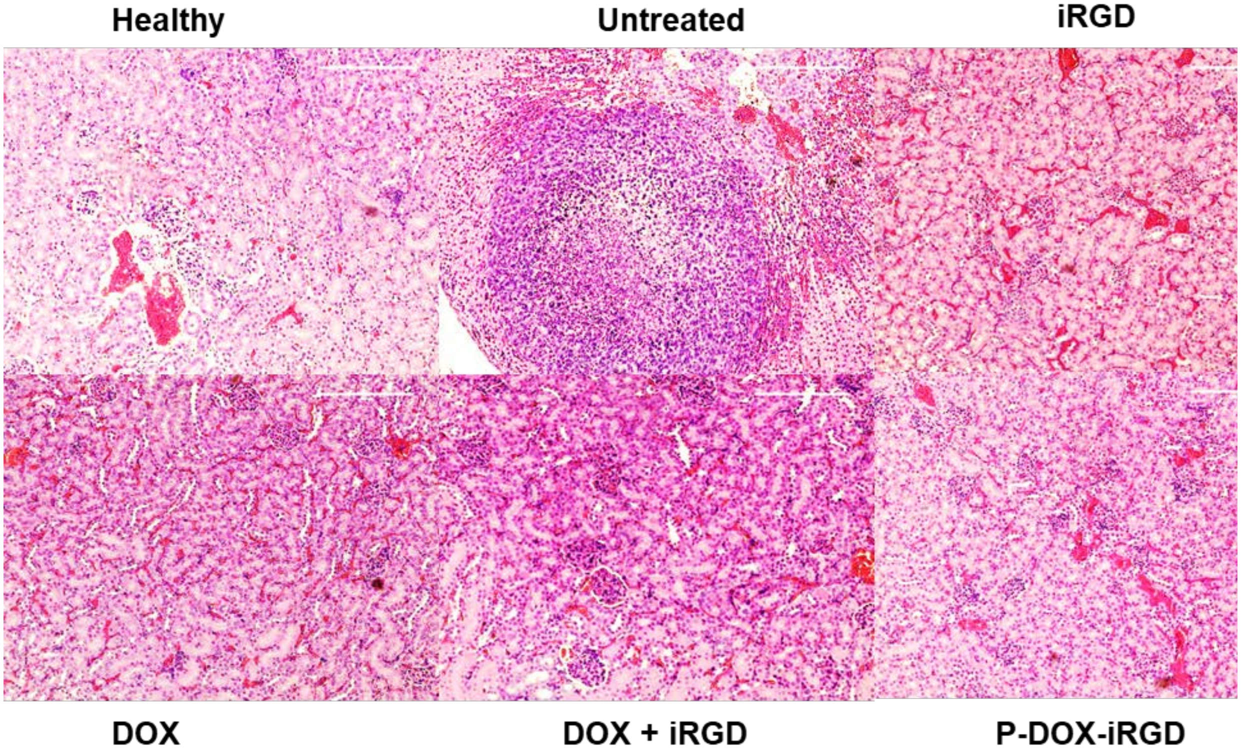

Healthy
Untreated
iRGD
DOX
DOX + iRGD
P-DOX-iRGD

(**B**)

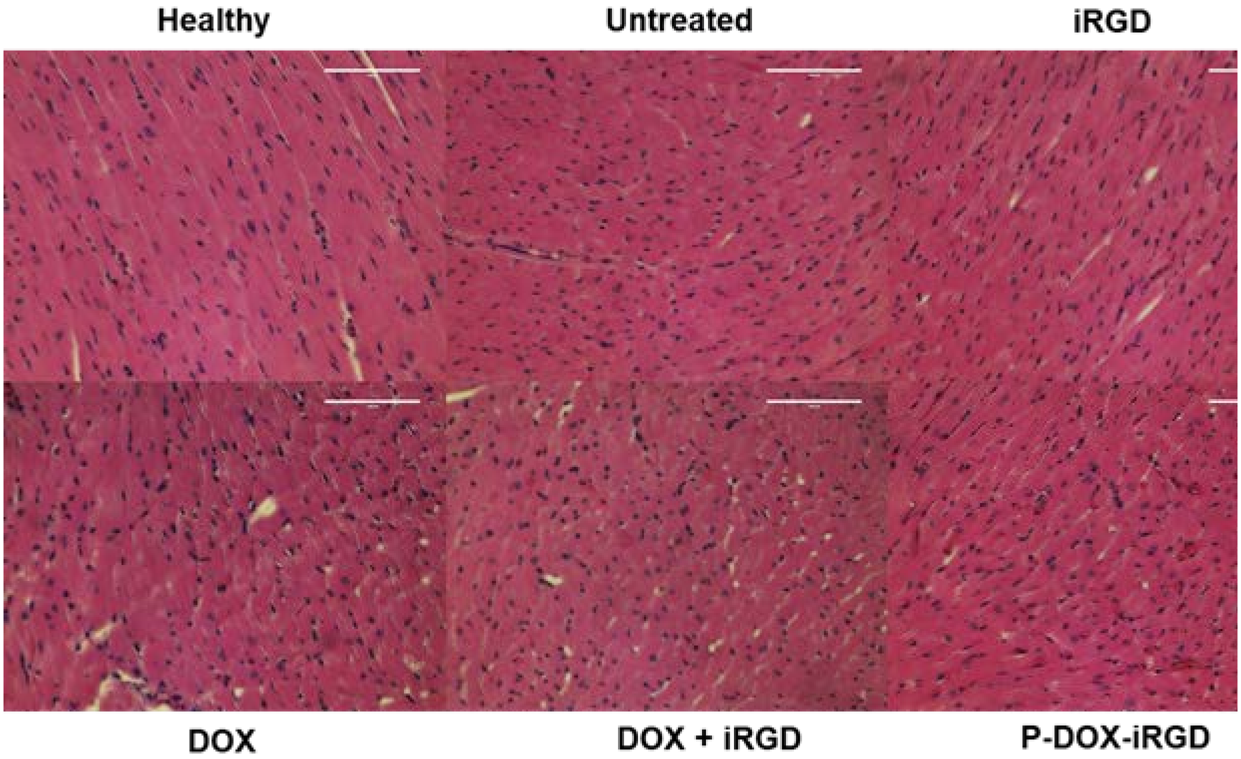

Figure S2C. Hematoxylin and eosin stains (H&E) of heart. Scale bar = 500 μm

(C)

Figure S2. (A) Average body weight change (Mean ± SD, n =8) of 4T1 tumor bearing mice. (B) Hematoxylin and eosin stains (H&E) of kidneys. Scale bar = 200 μm. (C) Hematoxylin and eosin stains (H&E) of heart.
